# COVID-19 Impact on Musculoskeletal Regenerative Medicine Research: Maintaining Lab Continuity

**DOI:** 10.3390/ijerph18116110

**Published:** 2021-06-05

**Authors:** Livia Roseti, Brunella Grigolo

**Affiliations:** IRCCS Istituto Ortopedico Rizzoli, SSD RAMSES, Via di Barbiano 1/10, 40136 Bologna, Italy; brunella.grigolo@ior.it

**Keywords:** regenerative medicine, COVID-19, control measures, risk assessment

## Abstract

Background: Research in the fields of musculoskeletal tissue engineering and regenerative medicine may suffer a slowdown during the ongoing COVID-19 pandemic emergency. This is likely to harm the development of new therapeutic strategies and their translation into the clinic in the long term. Recently, the need to maintain continuity in research activities in those fields has assumed even greater importance due to the accumulation of data concerning the effects of SARS-CoV-2 on the musculoskeletal system. This study is aimed at the identification of a series of safe handling practices against COVID-19 diffusion to apply in a research environment, thus allowing the maintenance of research lab activities. Methods: The control measures to apply to mitigate the COVID-19 risk were identified and categorized utilizing the Hierarchy of Controls. We also compared our analysis with that assessed before the pandemic to consider the additional risk of COVID-19. Results: Results highlighted that the most relevant implemented measures to control SARS-CoV-2 were based on protecting people through engineering (e.g., ventilation and social distancing), and administrative (e.g., hand sanitization, work shifts) measures or Personnel Protective Equipment, rather than eliminating hazards at the source (e.g., smart working). Conclusions: Work continuity in research labs during the COVID-19 emergency should be guaranteed by ensuring the protection of researchers in the workplace and considering the physical environment, the type of operators and work activity, and the proven ability of workers to face biological risks. The increased knowledge and awareness on lab’ risks should be useful to prevent and mitigate future viral outbreaks.

## 1. Introduction

The coronavirus disease 19 (COVID-19) pandemic, caused by the new virus “Severe Acute Respiratory Syndrome Coronavirus-2” (SARS-CoV-2) [1,2], largely impacted on several therapeutic strategies including regenerative medicine and the engineering of muculoskeletal tissues (cartilage, bone, tendons, ligaments, synovia, muscles) [3,4]. In the orthopedic routine, emergency traumas and oncologic interventions (major surgery) have been favored at the expense of treatments based on these new approaches (elective procedures) [5,6,7] that generally utilize cells, scaffolds, and growth factors that are potentially effective in triggering/increasing regeneration and healing and reducing inflammation [8]. As a final effect, the joint function may improve and pain may decrease. In such a way, there is a concrete possibility to slow down the development of osteoarthritis (OA) and then reverse its debilitating effects [9]. It has been observed that a delay in treatment of lesions leading to the development of OA due to the COVID-19 pandemic could result in an increased number of patients who will need joint replacement in the long term [5]. Added to this is the recent increase of data about the damaging effects of SARS-CoV-2 on the musculoskeletal system. Direct effects refer to the virus reaching, throughout the blood, the musculoskeletal tissues. Indirect effects refer to the prolonged patient inactivity due to hospitalization in intensive care units (ICU), imposed at-home lockdown, or remote working activities. Described symptoms can range from arthralgia/myalgia, myasthenia, and fatigue to severe conditions such as cachexia or sarcopenia [10,11,12]. Hence, it becomes even more important to investigate, alongside physical and rehabilitation therapies, regenerative strategies that may contribute to preserve or reduce musculoskeletal system damage and improve patient quality of life.

This pandemic has led to a global fund mobilization by governments and agencies to finance research projects to combat COVID-19 [13]. In many countries, not only new resources have been allocated but also several existing research funds have been re-oriented toward COVID-19 investigations [14]. In the meantime, several experimental studies that were already in progress in other fields, including the musculoskeletal one, have been mostly delayed due to the lockdowns/restrictions that kept researchers at home. Moreover, various pharmaceutical and biotech companies, capitalizing on their experience in engineering techniques such as in vitro models and drug delivery [15], have shifted their focus on the development of drugs or vaccines to treat people infected with SARS-CoV-2. This situation caused existing research projects to suffer a slowdown and made it more difficult to start new research in other fields such as regenerative medicine in orthopedics. The possibility of a long-term lack of investments in tissue engineering and regenerative medicine research is likely to harm the development of new therapeutic strategies and their translation into the clinic [16]. Therefore, it is important to maintain research activities in labs as long as anti-COVID-19 requirements are met [17].

Many agencies have issued detailed guidelines as to how to manage COVID-19 in different work settings. However, most documents have focused primarily on specific work settings such as industry (productive chain and R&D), hospitals (inpatient wards, emergency rooms), and clinical laboratories and are therefore tailored for a specific environment [18,19,20,21]. COVID-19 risk management in academic/hospital research laboratories may be underestimated and unregulated. This poses a problem since research lab settings present their challenges that also need to be addressed. Our lab, located in an Italian Orthopedic Institute which is considered a center of excellence for orthopedics and traumatology, conducts investigations aimed at the prevention and treatment of orthopedic diseases. Preclinical research activities are carried out in the field of tissue engineering, rheumatology, and additive manufacturing, with the aim of translation to the clinic and industrial applications. Similar to most research institutes, many teams collaborate sharing spaces, instruments, reagents, and laboratory materials. There are hardly single workstations in single offices/laboratories but rather open spaces.

In this study, we identified a series of safe handling practices against COVID-19 diffusion in a research lab located in a hospital environment, which was based on the current legislative framework, literature, and our own experience. We also compared our analysis with that assessed before the pandemic in order to consider the additional risk of COVID-19.

## 2. Materials and Methods

### 2.1. Working Group

A group of experienced professionals from our lab was constituted. Its composition was heterogeneous in terms of roles, competencies, knowledge, and experience. In particular, the lab supervisor, the health and safety risk expert, and several researchers were present. The researchers were biologists/biotechnologists with experience in musculoskeletal tissue engineering and regenerative medicine research. The interdisciplinary nature of the group enabled a better highlighting of potential issues from different points of view.

### 2.2. Literature and Regulation Review

A careful search and analysis of various guidance and literature documents were performed to identify documents describing risk assessment in a research lab environment and the mitigation measures to put in place.

### 2.3. Risk Assessment

The risk assessment protocol utilized to control COVID-19 spread was developed by integrating the O*NET database of the US Bureau of Labor of Statistics and data from the Italian working context that has been analyzed and released from the Italian Institute for insurance against accidents at work (Istituto Nazionale per l’Assicurazione contro gli Infortuni sul Lavoro, INAIL) and the National Institute of Statistics (Istituto Nazionale di Statistica, ISTAT) [22,23]. This model is based on three variables: exposure, proximity, and aggregation (Table 1).

The team decided not to follow a template for this assessment but proposed a procedure that is common in most institutions’ risk assessments. In that way, the information can be easily transferred and the method adjusted to the different organizations. The procedure proposed to manage COVID-19 risk encloses the steps described below.

#### 2.3.1. Context

Before undertaking the risk assessment, the context was defined in terms of areas, personnel, and activities. The team considered not only the research lab context but the whole institute, since the surrounding environment may affect a research lab’s hazards and risks. The Rizzoli Orthopedic Institute is a highly specialized hospital and research facility in the field of orthopedics and traumatology. It is also the venue of University teaching. The fact that The Rizzoli Orthopedic Institute comprises both treatment and research activity allows the results of scientific research to be easily translated into clinical practice. From a logistical point of view, it is organized in three close locations: the hospital giving patients medical and surgical care, the polyclinic providing specialist examinations, and the research center, which besides the labs houses also the management and the administrative offices. Connected to the research area, there is the animal facility, which is a unit that deals with housing and breeding animals and experiments on them in compliance with current regulations. Such an organization allows better communications between researchers and clinicians and the translation of research results into clinical practice.

**Figure 1:**
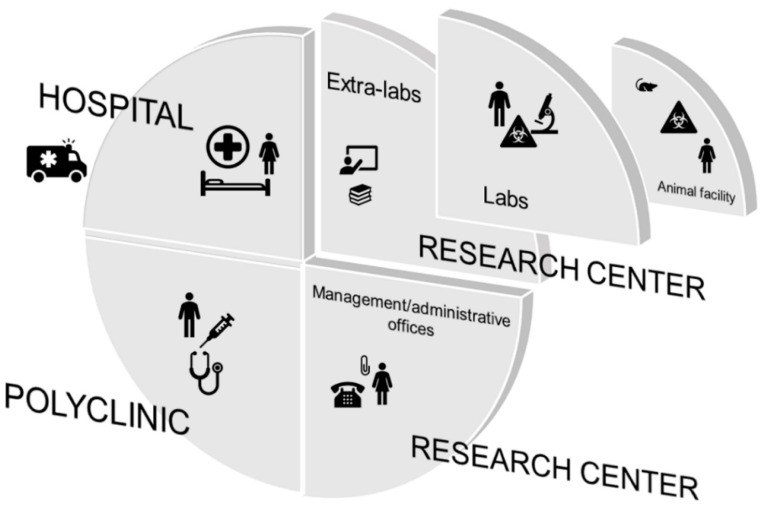
Sub-contexts from which to start for risk assessment: general, extra-lab, and research lab. The general sub-context is represented by the hospital, the polyclinic, and the management and administrative offices. What is defined as a “research laboratory” is made up of two different environments: extra-lab areas where bibliographic research, writing of scientific works, or projects meetings and teaching activities take place; and lab areas where experiments are carried out.

To simplify the analysis, the team split up such a complex context into three sub-contexts: general, extra-lab, and research lab. The general sub-context is represented by the hospital, the polyclinic, and the management and administrative offices. What is defined as a “research laboratory” is made up of two different environments: extra-lab areas where bibliographic research, writing of scientific works, or projects meetings and teaching activities take place; and lab areas where experiments are carried out (Figure 1).

Due to the proximity and continuous personnel passing between the three sub-contexts, the team identified for the researchers three levels of risk assessment corresponding to the identified sub-contexts: general, extra-lab, and (research) lab. Details are listed in Table 2 in terms of personnel and activities.

#### 2.3.2. Hazard

The source of exposition that may cause harm (COVID-19) was defined.

#### 2.3.3. Risk

The risks caused by the SARS-CoV-2 virus have been described.

#### 2.3.4. Existing vs. Additional Control Measures

The team considered what measures, if any, were already in place to control the biological risk and if they were adequate. Then, the group decided if more measures can be applied. As a final step, the team evaluated the biological risk containment measures already put in place before the pandemic in comparison with the addition emergency ones against SARS-CoV-2.

The control measures to apply to mitigate the COVID-19 risk were categorized utilizing the Hierarchy of Controls, which is a risk management tool used around the world. It can be described as a flow that goes from the most effective measure to the least: Elimination, Substitution, Isolation, Engineering controls, Administrative controls, and Personnel Protective Equipment (PPE) (Table 3) [24].

**Figure 2:**
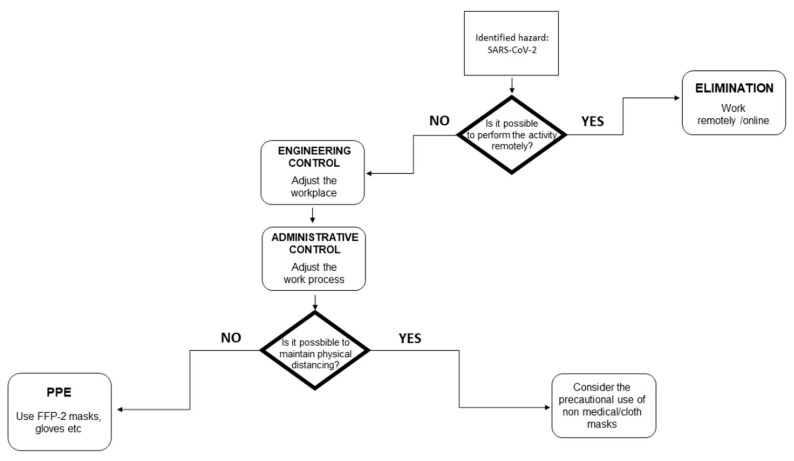
Decisional flowchart for the managing of the Hierarchy of Controls to mitigate SARS-CoV-2. The flowchart shows the steps as boxes and their order by connecting the boxes with arrows. The boxes with rhombus shapes are decisional steps.

The decisional flow utilized for the managing of the Hierarchy of Controls to mitigate SARS-CoV-2 is detailed in Figure 2. The first evaluation is the possibility to eliminate/substitute the hazard to mitigate the risk. Otherwise, engineering and administrative control measures should be applied. Then, there is a second decisional moment: the possibility of physical distancing. If not maintained, the use of PPE should be evaluated.

## 3. Results

### 3.1. Working Group

The team started working at the beginning of the pandemic, and since then, regular weekly meetings have occurred until the present moment. In this way, the group was able to check on the documental body finding new updates and elaborating specific recommendations.

### 3.2. Literature and Regulation Review

Focusing on guidelines/reviews/analyses concerning COVID-19 risk in research labs, we found four types of documents as listed in Table 4, together with their characteristics. As expected, documents were generally local or tailored for a specific environment (i.e., University campus) [25,26,27,28,29,30,31].

### 3.3. Risk Assessment

#### 3.3.1. Hazard

**Figure 3:**
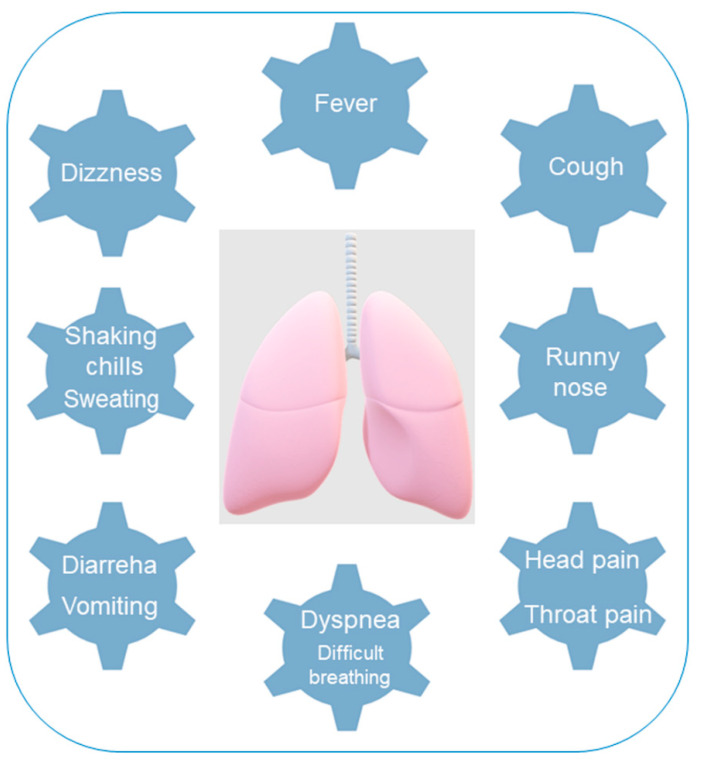
Symptoms of COVID-19. The figure graphically represents the most reported symptoms of COVID-19.

The hazard to consider is the SARS-CoV-2 virus, which is responsible for the infection and the development of the COVID-19 disease, which has had a wide range of symptoms reported: from mild to severe illness (and death) (Figure 3) [32,33].

#### 3.3.2. Risk

SARS-CoV-2 risks of transmission identified based on guidance documents and literature are droplets, aerosol, contaminated surfaces, fomites, and animal-to-human (Table 5) [34,35].

#### 3.3.3. Existing vs. Additional Control Measures

**Figure 4:**
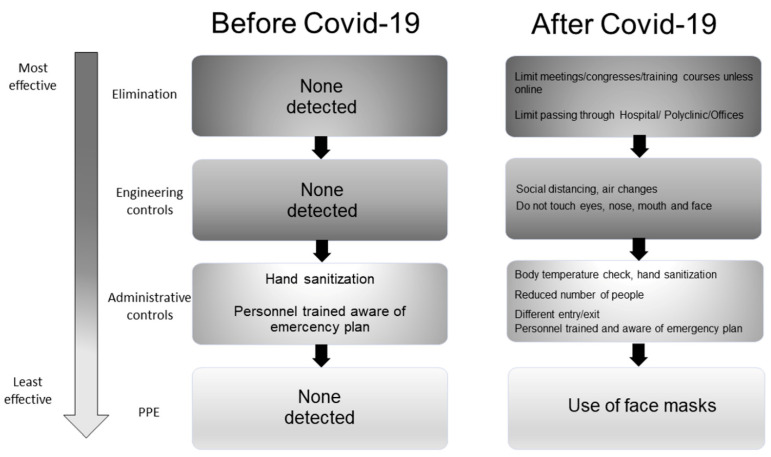
Hierarchy of Controls to identify the “general” best practices for COVID-19 control. Depicted with a vertical arrow list, the most effective controls are on the top side, whereas the least effective controls are on the bottom. Left part: existing measures; Right part: emergency measures.

**Figure 5:**
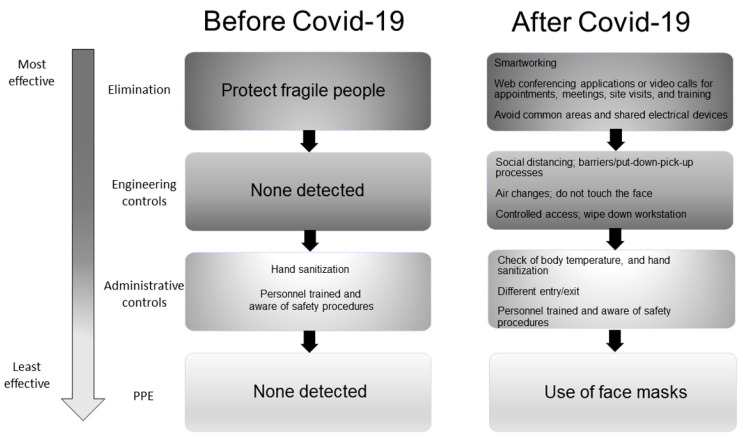
Hierarchy of Controls to identify the “extra-lab” best practices for COVID-19 control. Depicted with a vertical arrow list, the most effective controls are on the top side, whereas the least effective controls are on the bottom. Left part: existing measures; Right part: emergency measures.

**Figure 6:**
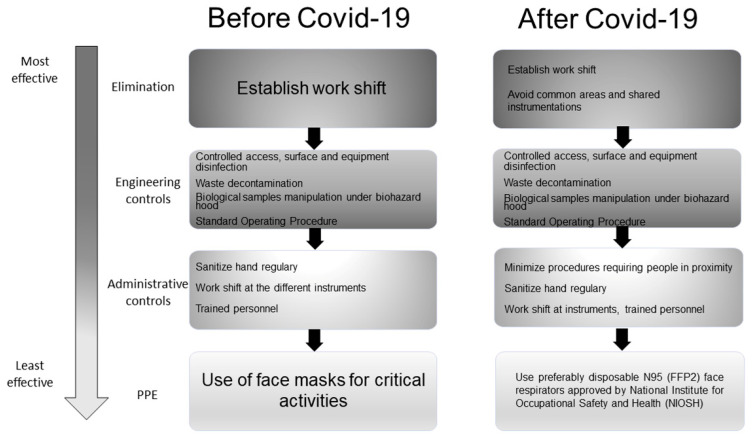
Hierarchy of Controls to identify the “research-lab” best practices for COVID-19 control. Depicted with a vertical arrow list, the most effective controls are on the top side, whereas the least effective controls are on the bottom. Left part: existing measures; Right part: emergency measures.

The general, extra-lab, and lab existing (before COVID-19) and adopted (after COVID-19) control measures against biological risk are listed in Figure 4, Figure 5, and Figure 6, respectively. Figure 4 describes the risks taken by the researchers when they were located in the hospital, polyclinic, or management/administrative offices. Before the pandemic (Figure 4, left part), only some administrative controls have been already applied such as hand sanitizing when entering the wards. Moreover, personnel had been already trained also on the emergency/incident plan of such areas. The COVID-19 pandemic has raised the need to activate the whole Hierarchy of Controls (Figure 4, right part). In particular, researchers were invited to consider if it was necessary to be in those areas and limit interactions (meetings, congresses, training courses) with the hospital/polyclinic personnel by utilizing online meeting and conferencing tools (Elimination). If in-person presence was inevitable, social distancing, air changes, and avoiding touching the eyes, noise, mouth, and face (Engineering Controls) were a must. When entering such areas, researchers were checked for body temperature and monitored for possible gatherings and the respect of different entry/exit paths (Administrative Controls). Researchers were also requested to use face masks when circulating (PPE). Substitution measures were non-detected in both cases.

Figure 5 describes the risks taken by the researchers when they are in the “extra lab” environment, i.e., staff, meeting, and training rooms, warehouse, and toilets. Before the pandemic (Figure 5, left part), there was the possibility of agile working only for fragile people (elimination) so to allow them to receive adequate assistance/therapy. Some administrative controls have been already applied such as hand sanitizing (SafeHands WHO campaign) and the awareness (by training) of the emergency plan. The pandemic has raised the need to activate a plethora of specific containment measures (Figure 5, right part). The Elimination measure to work remotely has been extended to all personnel (staff and students) when possible (to perform non-lab activities such as paper/project writing and reviewing). Web conferencing applications or video calls were endorsed for appointments, meetings, site visits, and training courses. Recommendations were also to avoid common areas and shared electrical devices. Engineering measures consisted of social distancing and the use of physical barriers or put-down–pick-up processes where necessary and when possible; air changes; avoiding touching the eyes, nose, mouth, and face; controlled access; and wiping down workstations before and after use (PC, printers, furniture) and toilets. Adjunctive administrative controls were to check body temperature and establish different entry/exit pathways when possible. The use of face masks was mandatory. Substitution measures were non-detected in both cases.

Figure 6 describes the risks taken by the researchers when they are in the lab environment. Before the pandemic (Figure 6, left part), the biological containment had been already put in place, as highlighted by the several applied control measures. In particular, work shifts had been already established (Elimination); access to critical areas was controlled; surfaces and equipment were disinfected before and after use, and the use of the appropriate method for waste decontamination was foreseen. Biological samples were manipulated under a biohazard hood. Safety clear-cut directions and instructions already existed as written Standard Operating Procedure (Engineering controls). The administrative controls that have been already applied were hand sanitizing (SafeHands WHO campaign), work shift at the different instruments, and personnel training. The use of face masks was recommended for critical activities. The emergency has focused the attention on viral contagion protection (Figure 6, right part). In addition to planned work shifts, recommendations were to avoid occupies/common areas and shared instrumentation (Elimination). Along with controlled access to critical areas and the use of an appropriate method for waste decontamination, the disinfection of surface and equipment before and after use utilizing disinfectants with proven activity against enveloped viruses was foreseen. Biological samples were manipulated under biohazard hoods and safety clear-cut directions and instructions in the form of a written Standard Operating Procedure were applied as well as before the pandemic (Engineering Controls). Many administrative controls that have been already applied were as well confirmed such as hand sanitizing, work shift at the different instruments, and personnel training (Administrative Control). The use of disposable face masks was recommended. Substitution measure were non-detected in both cases.

## 4. Discussion

The health, demographic, social, epidemiological, economic, and occupational world contexts have been profoundly and suddenly changed by the COVID-19 pandemic [36]. In such a situation, health system employees continued their job for obvious reasons. Hospital/academic research labs represent a heterogeneous working environment whose activity needs to be pursued; otherwise, the development of new therapeutic strategies and their translation into the clinic may be hampered with negative consequences on health and quality of life of patients in the long period. The laboratory supervisor has the responsibility to monitor the safe conduct of all researchers in the lab. Therefore, staff who are aware of the importance of safety and capable of identifying and controlling the risks is fundamental for the prevention of infections.

While the risk assessment of COVID-19 receives significant regard in the clinical setting, there is still limited attention—In terms of relevant literature—for controlling and mitigating the risks within Hospital/Academic research labs. Moreover, the simple adoption or application of standardized industrial best practices may be difficult due to the nature of work and personnel. Work activities in research labs are indeed diversified in terms of types, activity, personnel, and research projects. Hence, we considered it appropriate to identify a series of safe handling practices against COVID-19 diffusion to apply in a research lab. The methodology utilized for the COVID-19 risk assessment was chosen especially because it is based on a model already adjusted to the Italian working context. Furthermore, it allows synthesizing and integrating the areas of risk and the corresponding impacts related to the parameters of exposure, proximity, and aggregation. The team decided not to follow a template for risk assessment but proposed a procedure that is already common in most institutes. The lack of quantitative ratings may lead to oversimplifying the complexity or volatility of the risks, but the information can be easily transferred and the method adjusted to the different organizations. Our own experience helped us confront measures that have been already put in place before the COVID-19 pandemic and upgraded controls that needed to be implemented to further minimize the risks. In order to perform a full risk assessment, we considered not only the activity in the research lab context but in the whole institute, since the surrounding environment may affect hazards and risks. Therefore, we identified three sub-contexts to be all analyzed from the researcher’s point of view, but separately, since researchers can carry out different aspects of their work: general, extra-lab, and research lab (see Table 4). Overall, we found that the most relevant measures to control SARS-CoV-2 were based on protecting people through engineering (e.g., ventilation and social distancing), and administrative (hand sanitization) measures or PPE, rather than eliminating hazards at the source (e.g., smart working). Flexible working such as teleworking and staggered hours could be applied only partially i.e., when the researchers are dedicated to paper/project writing, literature search, or meetings. Substitution measures were not applied.

At the time the researchers were in the hospital, polyclinic, or management/administrative offices before the pandemic, they had to apply only some administrative controls such as hand sanitization and different entries and exits; moreover, they have been already trained and aware of the institute’s emergency/incident plan. The contacts between clinical and researchers were encouraged, since translational activities were considered one of the strengths of the institute. The emergency has raised the need to activate the whole Hierarchy of Controls of specific containment measures while still considering clinic–research integration as strategic. A recommendation was made to limit in-person interaction with the hospital/polyclinic personnel, preferring web meeting applications, and, if inevitable, applying social distancing.

The extra-lab environment—staff/meeting rooms—and activities—reading, writing, and literature searching—were separated from the “real labs”, and a few administrative controls have been already applied. The possibility of agile working was authorized only for fragile people. When the pandemic started, an issue arose that in general, single rooms were rare, while there were open spaces with people working. To contain the virus spread, social distancing, physical barriers, personal hygiene, and the use of face masks were recommended [37]. The possibility of smart working has been extended to all personnel when possible.

The real experimental work must be performed on-site with precautions. Before the pandemic, in the labs processing biological samples, the biological risk had been already faced as highlighted by the several ongoing control measures: work shift, biohazard hoods, controlled access, Standard Operative Procedures, and trained personnel. The COVID-19 pandemic pushed each lab to decide if the existing biological risk assessment also covers SARS-CoV-2 issues or if it must be implemented with new evaluations. In particular, the attention has been focused on contagion protection (social distancing, use of specific disinfectants).

With this paper, we propose a series of safe handling practices against COVID-19 diffusion to apply in research labs not only as an immediate response to the pandemic emergency but also ensuring continuity of the activities in the long term. It must be underlined that this situation is heterogeneous among countries (both for policies and for citizen behaviors) and constantly evolving, such as for the new virus variants and the recent availability of approved anti-COVID-19 vaccines. To keep up with all those changes, specific guidelines are continuously revised accordingly. Researchers must develop an enhanced level of awareness on health and safety guidelines and their subsequent amendments.

Thanks to the international coordination and collaboration among studies, pharmaceutical companies, regulators, and governments, the recent possibility of vaccination represents a turning point in the management of the pandemic. Even if no vaccine is 100% effective, preventing access to host cells makes the virus unable to replicate [38]. Within the Hierarchy of Controls, vaccination represents the first and most effective “Elimination” control.

## 5. Conclusions

Work continuity in research labs during COVID-19 emergencies should be guaranteed by ensuring the protection of researchers in the workplace and considering the physical environment, the type of operators and work activity, and the proven ability of workers to face biological risks. We believe that after such an experience, it will not possible for researchers to completely resume “normal” activities, and that increased knowledge and awareness on risks and an improvement of the safety of the system will be critical to prevent/mitigate future viral outbreaks [39].

In the case of research on musculoskeletal regenerative medicine and tissue engineering, a prolonged interruption may impair patients’ health. Recently, this issue has gained greater importance due to the accumulation of data concerning the effects of SARS-CoV-2 on the musculoskeletal system.

## Figures and Tables

**Table 1:** Critical variable to consider for SARS-CoV-2 risk assessment.

Variable	Definition
Exposure	The probability of contagion during work activities
Proximity	Physical distancing during working periods
Aggregation	Contact with other subjects in addition to colleagues

**Table 2:** Levels of risks assessment to consider for sub-context, operator, and activity.

Level of Risk Assessment	Sub-Context	Operator	Activity
General	Hospital, polyclinic	Orthopedics, rheumatologists, radiologists, oncologists, other specialists, nurses, physiotherapists, pharmacists, teachers, social workers, psychologists, researchers	Clinical and surgical activities carried out on patients
	Management and administrative offices	Technical and administrative staff	Administrative and managing activities
Extra-Lab	Staff, meeting, training rooms	Researchers (biologists, biotechnologists, technicians, bioengineers, physicians, chemists)	Project/manuscript design, writing and submission bibliographic research, meetings, teaching
	Warehouse	Warehouse workers	Materials handling
	Toilets	Cleaners	Cleaning
Lab	Lab rooms: cell culture, histochemistry immunohistochemistry, molecular biology, 3D printing	Researchers (biologists, biotechnologists, technicians, bioengineers, physicians, chemists)	Cell culturing, immunohistochemistry, molecular biology, 3D printing
	Animal facility	Veterinarians	Animal caring and surgery

**Table 3:** Hierarchy of Controls representation.

Control Measure	Definition
Elimination	Physically remove the hazard
Substitution	Replace the hazard
Engineering controls	Isolate people from the hazard
Administrative controls	Change the way people work
PPE	Protect the worker with PPE

**Table 4:** Guidelines/reviews/analyses concerning COVID-19 risk in research labs.

Type of Document	Characteristic
Institutional guidelines for research facilities [25,26,27]	Legitimate at state level
Guidelines for research facilities [28]	Legitimate at local level
University guidelines for research facilities [29]	Tailored for each University
Enterprise guidelines for R&D facilities [30,31]	Specific for R&D environment

**Table 5:** SARS-CoV-2 risks of transmission.

Risk	Transmission
Contact	Direct (through secretions), indirect
Respiratory droplet (>5–10 μm)	Proximity with an infected person coughing, sneezing, talking, or singing
Airborne	Aerosols (<5 μm droplets) that remain infectious when suspended in air over long times and distances
Fomite	Touching of fomites (surfaces or objects contaminated with secretions or droplets from an infected person), followed by touching the mouth, nose, or eyes
Animal-to-human	SARS-CoV-2 is most closely related to beta coronaviruses in bats; the role of intermediate host in facilitating transmission in humans remains unclear
